# Efficacy of remote ischaemic preconditioning on outcomes following non-cardiac non-vascular surgery: a systematic review and meta-analysis

**DOI:** 10.1186/s13741-023-00297-0

**Published:** 2023-04-10

**Authors:** Aikaterini Papadopoulou, Matthew Dickinson, Theophilus L. Samuels, Christian Heiss, Lui Forni, Ben Creagh-Brown

**Affiliations:** 1 Department of Anesthesia, King’s College Hospital, Denmark Hill, London, SE5 9RS UK; 2 Department of Anesthesia, Royal Surrey County Hospital, Guildford, UK; 3 Department of Critical Care, Surrey and Sussex Healthcare NHS Trust, Redhill, UK; 4 Vascular Department, Surrey and Sussex Healthcare NHS Trust, Redhill, UK; 5 Department of Clinical and Experimental Medicine, University of Surrey, Guildford, UK; 6 Department of Critical Care, Royal Surrey County Hospital, Guildford, UK

**Keywords:** Ischaemic preconditioning, Non-cardiac surgery, Postoperative acute kidney injury, Postoperative morbidity, Postoperative myocardial injury, Postoperative troponin

## Abstract

**Background:**

Remote ischaemic preconditioning (RIPC) has been investigated as a simple intervention to potentially mitigate the ischaemic effect of the surgical insult and reduce postoperative morbidity. This review systematically evaluates the effect of RIPC on morbidity, including duration of hospital stay and parameters reflective of cardiac, renal, respiratory, and hepatic dysfunction following non-cardiac non-vascular (NCNV) surgery.

**Methods:**

The electronic databases PubMed, Embase, and the Cochrane Central Register of Controlled Trials (CENTRAL) were searched from their inception date to November 2021. Studies investigating the effect of local preconditioning or postconditioning were excluded. Methodological quality and risk of bias were determined according to the Revised Cochrane risk-of-bias tool for randomised trials (RoB 2). Calculation of the odds ratios and a random effects model was used for dichotomous outcomes and mean differences or standardised mean differences as appropriate were used for continuous outcomes. The primary outcomes of interest were cardiac and renal morbidity, and the secondary outcomes included other organ function parameters and hospital length of stay.

**Results:**

A systematic review of the published literature identified 36 randomised controlled trials. There was no significant difference in postoperative troponin or acute kidney injury. RIPC was associated with lower postoperative serum creatinine (9 studies, 914 patients, mean difference (MD) - 3.81 µmol/L, 95% confidence interval (CI) - 6.79 to - 0.83, *p* = 0.01, *I*^2^ = 5%) and lower renal stress biomarker (neutrophil gelatinase-associated lipocalin (NGAL), 5 studies, 379 patients, standardized mean difference (SMD) - 0.66, 95% CI - 1.27 to - 0.06, *p* = 0.03, *I*^2^ = 86%). RIPC was also associated with improved oxygenation (higher P_a_O_2_/F_i_O_2_, 5 studies, 420 patients, MD 51.51 mmHg, 95% CI 27.32 to 75.69, *p* < 0.01, *I*^2^ = 89%), lower biomarker of oxidative stress (malondialdehyde (MDA), 3 studies, 100 patients, MD - 1.24 µmol/L, 95% CI - 2.4 to - 0.07, *p* = 0.04, *I*^2^ = 91%)) and shorter length of hospital stay (15 studies, 2110 patients, MD - 0.99 days, 95% CI - 1.75 to - 0.23, *p* = 0.01, *I*^2^ = 88%).

**Conclusions:**

This meta-analysis did not show an improvement in the primary outcomes of interest with the use of RIPC. RIPC was associated with a small improvement in certain surrogate parameters of organ function and small reduction in hospital length of stay. Our results should be interpreted with caution due to the limited number of studies addressing individual outcomes and the considerable heterogeneity identified.

**Trial registration:**

PROSPERO CRD42019129503.

**Supplementary Information:**

The online version contains supplementary material available at 10.1186/s13741-023-00297-0.

## Introduction

Postoperative morbidity, as defined by the National Surgical Quality Improvement Programme (NSQIP), not only affects short-term outcomes resulting in a prolonged hospital stay and delayed adjunct treatment, but there is also evidence that it is associated with longer-term effects including survival and disease recurrence (Artinyan et al. 2015; Aoyama et al. 2015; Khuri et al. 2005). Following the body cavity surgery, oxygen consumption increases from approximately 3.5 ml/kg/min in the resting state to 5 ml/kg/min (Minto and Biccard 2014). If this increased oxygen demand cannot be met, the resultant supply/demand imbalance may result in tissue ischaemia (Minto and Biccard 2014). Restoration of the blood flow to an ischaemic organ results in an inflammatory response that may augment tissue injury in excess of that produced by ischaemia alone. Ischaemic preconditioning describes a brief episode of ischaemia that initiates a response which protects organs from sustained ischaemic events and as such has the potential to attenuate the ischaemic and reperfusion impact of the surgical insult (Zarbock et al. 2020).

The mechanism underlying remote ischaemic preconditioning (RIPC) is not completely understood, but likely involves both neuronal and humoral factors that result in vagally mediated cardioprotection and nitric oxide-induced mitochondrial protection, respectively (Hausenloy et al. 2015; Sivaraman et al. 2015; Wu et al. 2018).

The technique of RIPC has been studied mostly in cardiac and vascular surgery and a Cochrane systematic review concluded that, although RIPC did not improve mortality, myocardial infarction, or stroke, it decreased the release of troponin following cardiac surgery (Benstoem et al. 2017). RIPC has been studied less extensively in non-cardiac non-vascular (NCNV) surgery, and therefore, we performed a systematic review and meta-analysis of the effect of RIPC on postoperative morbidity in this group of patients.

## Methods

We performed a systematic review and meta-analysis in accordance with the Preferred Reporting Items for Systematic Reviews and Meta-analyses (PRISMA) statement criteria and the Cochrane collaboration recommendations. The protocol was registered with Prospero under the registration number CRD42019129503.

### Search strategy

The electronic databases PubMed, Embase, and the Cochrane Central Register of Controlled Trials (CENTRAL) were searched from their inception date to November 2021. The search terms used were ischemic condition* or ischemic precondition* or ischaemic condition* or ischaemic precondition*(title). There were no language restrictions. Google search engine was also searched for additional publications.

### Inclusion and exclusion criteria

The search results were limited to randomised controlled trials investigating the use of RIPC prior to NCNV surgery. Studies investigating the effect of local preconditioning (direct interruption of the arterial blood supply to the organ undergoing the surgical intervention) or postconditioning (interruption of the blood supply after the completion of the surgical procedure) were excluded. Studies where the RIPC was applied in one subject and the outcome of interest was investigated in a different subject (i.e., studies where the RIPC was applied to the organ donor and the outcomes were measured in the organ recipient) were also excluded.

### Outcome parameters

The primary outcome of interest was postoperative cardiac morbidity, as defined by the incidence of myocardial injury as well as postoperative troponin levels, and renal morbidity characterized by the incidence of acute kidney injury (AKI), postoperative creatinine and glomerular filtration rate (GFR) values, and the renal stress biomarker neutrophil gelatinase-associated lipocalin (NGAL), a glycoprotein of the lipocalin superfamily that is produced by the kidney within hours of an ischaemic insult and its level correlates with the severity of AKI. The outcomes were chosen for better capture and overall assessment of the effects of RIPC on renal parameters.

The secondary outcomes of interest were chosen based on outcomes studied in the literature and included other metrics of organ dysfunction or morbidity, namely, respiratory, hepatic, markers of inflammation and oxidative stress, and length of hospital stay. Specific indices investigated were the ratio of arterial oxygen partial pressure to fractional-inspired oxygen (P_a_O_2_/F_i_O_2_), arterial to alveolar partial pressure of oxygen (P_a_O_2_/P_A_O_2_), and alveolar-arterial partial pressure of oxygen difference (P_A_O_2_-P_a_O_2_). Liver function tests included alanine aminotransferase (ALT), aspartate aminotransferase (AST) and bilirubin, and markers of inflammation and oxidative stress included interleukin 6, tumor necrosis factor a (TNF-a), and malondialdehyde (MDA). The adverse effects of remote ischaemic preconditioning were also investigated.

### Study selection

The two authors (AP and MD) independently screened the titles and abstracts returned by the search against the inclusion criteria. Full-text articles were obtained for the abstracts that met the inclusion criteria and were examined by the same two authors who reached a decision about inclusion. The reason for the exclusion of any full-text article was noted.

### Data extraction

Data were extracted by one author (AP) and were cross-checked by the other authors. We extracted information about the general characteristics of each study (author, date, type of study) the participants (characteristics of the population and type of surgery), the intervention (place of tourniquet placement, number of cycles, and duration of each inflation), and the outcomes. For dichotomous outcomes, we extracted the number of events that occurred, and for continuous outcomes, the mean values and standard deviations. Where only a graph was available, data were extracted using the WebPlotDigitiser tool (WebPlotDigitizer - Copyright 2010-2020 Ankit Rohatgi 2020). Where the result was reported as mean and confidence intervals, the standard deviation was obtained by dividing the length of the confidence interval by 3.92 and then multiplying by the square root of the sample size (7.7.3.2 Obtaining standard deviations from standard errors and. 2021) Where the result was reported as median [interquartile range], the median was used as the mean and the standard deviation was obtained by dividing the interquartile range by 1.35 (7.7.3.5 Medians and interquartile ranges 2021).

### Assessment of methodological quality

Methodological quality and risk of bias were determined according to the Revised Cochrane risk-of-bias tool for randomised trials (RoB 2).

### Statistical analysis

A meta-analysis of the outcomes of interest was conducted using R (version 4.0.1) (R: the R project for statistical computing 2021). Calculation of the odds ratios and a random effects model was used for dichotomous outcomes and mean differences or standardised mean differences as appropriate were used for continuous outcomes. The *I*^*2*^ statistic was used to measure heterogeneity and values greater than 50% were considered to indicate significant heterogeneity. The GRADE approach was used to assess the quality of the evidence.

## Results

### Literature search and selection

**Fig. 1:**
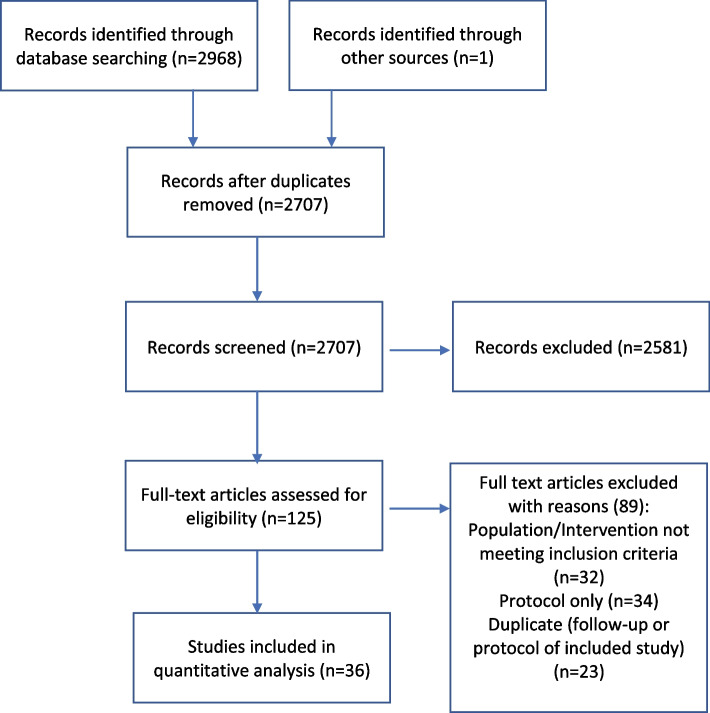
PRISMA flow diagram

The systematic literature search identified 2707 relevant publications. Of the 125 full-text articles assessed for eligibility, 36 were suitable to be included in the systematic review (Supplemental Table 1). The study selection process is shown in the PRISMA flow diagram (Fig. 1).

### RIPC technique

RIPC was applied to the upper limb in 17 studies, and the lower limb in 18 studies and either the arm or calf in 1 study (Supplemental Table 1). Of the 17 studies that applied RIPC to the upper limb, 12 showed at least one positive outcome (71%), whereas 15 of the 18 studies that applied RIPC to the lower limb had at least one positive outcome (83%), *p* = 0.443. Of the 27 studies that reported at least one positive outcome, 8 included the use of intravenous (propofol) maintenance of anaesthesia, 12 used regional or volatile maintenance, and 7 did not specify.

### Postoperative cardiac outcomes

**Fig. 2:**
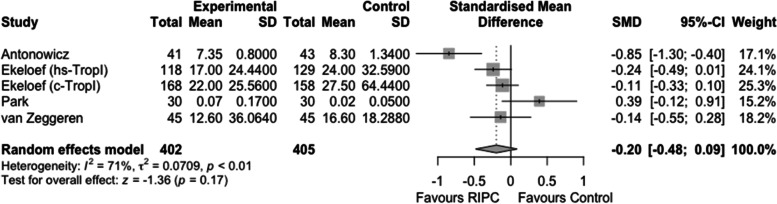
Meta-analysis comparing the effects of remote ischaemic preconditioning (RIPC) versus control on serum troponin in patients undergoing non-cardiac non-vascular surgery

A systematic review of the literature on the effect of RIPC on postoperative troponin following NCNV surgery returned 4 studies that included elective abdominal, orthopedic, and emergency hip fracture surgery (Antonowicz et al. 2018; Ekeloef et al. 2019; Park et al. 2018; Zeggeren et al. 2021). Two of the studies recorded the level of troponin on the first postoperative day, one recorded the peak troponin within the first 48 h, and one the peak troponin within the first 4 postoperative days. Antonowicz et al. and van Zeggeren et al. measured high-sensitivity troponin-T (hs-TropT), Park et al. measured cardiac troponin I (cTropI) and Ekeloef et al. changed from cTropI to high-sensitivity troponin-I (hs-TropI) during the study. There was no statistically significant difference between the intervention and control groups (standardised mean difference (SMD) - 0.2, 95% confidence interval (CI) - 0.48 to 0.09, *I*^*2*^ = 71%) (Fig. 2).

The incidence of postoperative myocardial injury was reported by 3 studies. However, the definition differed amongst the studies and, given the heterogeneous patient populations, the data were not pooled into a meta-analysis (Antonowicz et al. 2018; Ekeloef et al. 2019; Zeggeren et al. 2021). Antonowicz et al. recruited 84 patients undergoing elective abdominal surgery. Using a cutoff of peak hs-TropT in the first 72 h > 5 ng/L, the patients in the RIPC group had lower, though not statistically significant, incidence of perioperative myocardial injury (68 vs 81%, *p* = 0.211). Ekeloef et al. recruited 573 patients with a history of cardiovascular disease undergoing hip fracture surgery. The outcome changed from cTropI to hs-TropI during the study. The incidence of myocardial injury, defined as peak cTropI or hs-TropI of > 99th centile upper reference limit, was significantly lower in the RIPC group, 20 vs 31%, *p* = 0.002. Included in logistic regression analysis, RIPC was an independent variable associated with a lower risk of myocardial injury. Van Zeggeren et al. included 90 patients undergoing elective pancreatic surgery. Myocardial injury was defined as a rise in hs-TropT of at least 14 ng/L from the baseline and the incidence was 29 vs 40%, *p* = 0.375 in the intervention vs control group.

### Postoperative renal outcomes

**Fig. 3:**
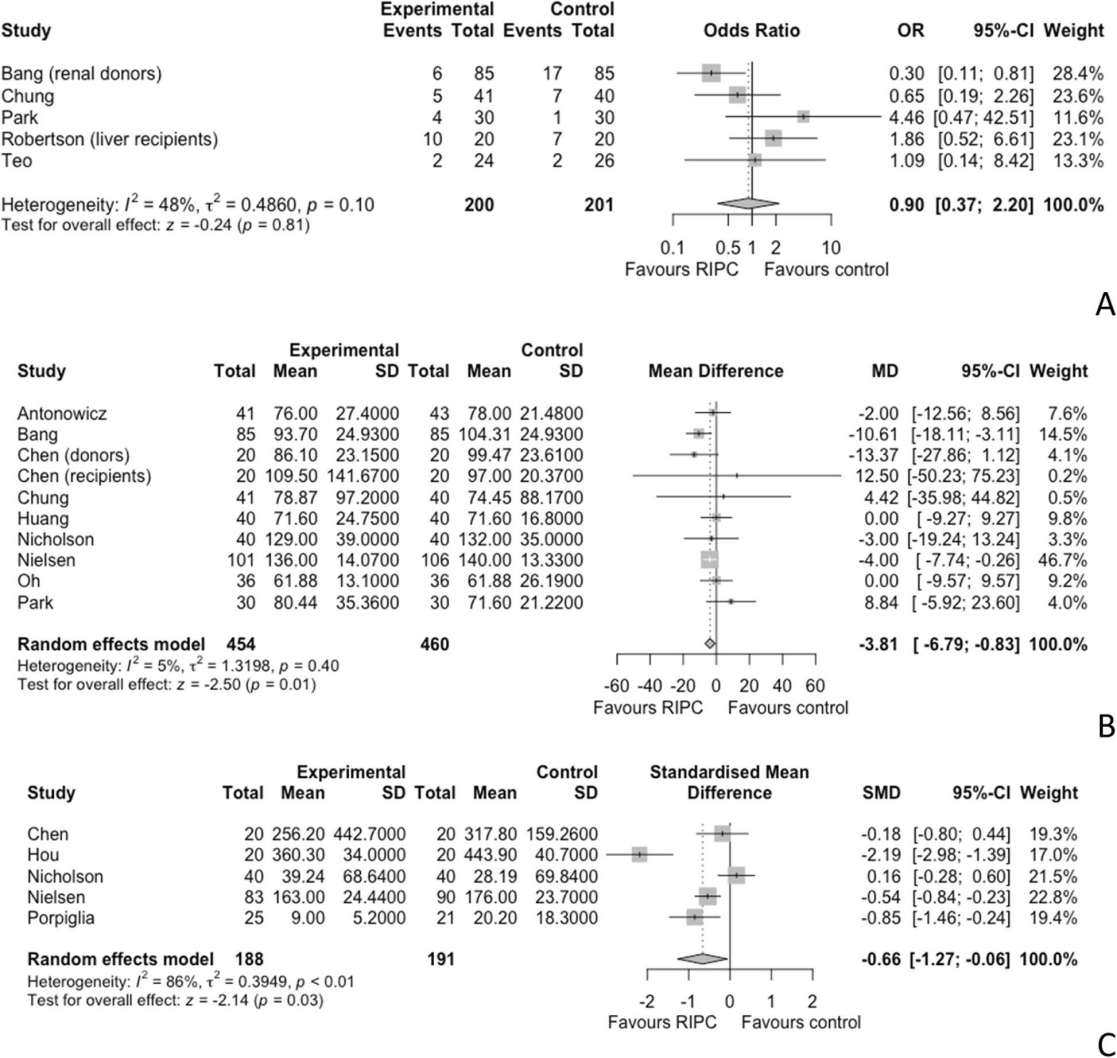
Meta-analysis comparing the effects of remote ischaemic preconditioning (RIPC) versus control on postoperative acute kidney injury (AKI) (**A**), serum creatinine (**B**), and neutrophil gelatinase-associated lipocalin (NGAL) (**C**) in patients undergoing non-cardiac non-vascular surgery

The incidence of postoperative acute kidney injury (AKI) was reported in 5 studies that included the following patient groups: patients undergoing nephrectomy, liver resection, orthopedic surgery, and liver transplant recipients (Park et al. 2018; Bang et al. 2019; Robertson et al. 2017; Teo et al. 2020; Chung et al. 2021). Three of the studies used the KDIGO criteria for the definition of AKI, one used the AKIN criteria and one used change in serum creatinine. The total number of participants was 547, and the incidence of AKI was 13.5 vs 16.92% in the intervention and control groups, respectively, *p* = 0.81 (Fig. 3A).

Values of postoperative serum creatinine were available from 9 studies. Random effects meta-analysis showed a lower postoperative serum creatinine in the intervention group, mean difference (MD) - 3.81 µmol/L, 95% CI - 6.79 to - 0.83, *p* = 0.01, *I*^2^ = 5%) (Fig. 3B) (Antonowicz et al. 2018; Park et al. 2018; Bang et al. 2019; Chung et al. 2021; Chen et al. 2013; Huang et al. 2013; Nicholson et al. 2015; Nielsen et al. 2019; Oh et al. 2017).

Postoperative GFR values were documented in 7 studies. Three of the studies evaluated GFR using technetium (99Tcm)-diethylene triamine pentacetic acid (DTPA) renal scintigraphy, one study used chrome-ethylenediamine tetraacetic acid (Cr-EDTA) and three studies used estimated GFR. There was no difference between the intervention and control groups (MD 1.22 mL/min/1.73 m^2^, 95% CI - 0.65 to 3.09, *p* = 0.20) (Supplemental Fig. 1) (Bang et al. 2019; Chung et al. 2021; Huang et al. 2013; Nicholson et al. 2015; Nielsen et al. 2019; Hou et al. 2017; MacAllister et al. 2015).

Five studies investigated the effect of RIPC on postoperative neutrophil gelatinase-associated lipocalin (NGAL), 2 following laparoscopic partial nephrectomy and 3 following renal transplant surgery with RIPC being performed on the recipients (Chen et al. 2013; Nicholson et al. 2015; Nielsen et al. 2019; Hou et al. 2017; Porpiglia et al. 2018). Two studies reported urine NGAL and three reported plasma NGAL. Postoperative NGAL was lower in the intervention compared to the control group SMD - 0.66, 95% CI - 1.27 to - 0.06, *p* = 0.03, *I*^*2*^ = 86% (Fig. 3C).

### Postoperative respiratory outcomes

**Fig. 4:**
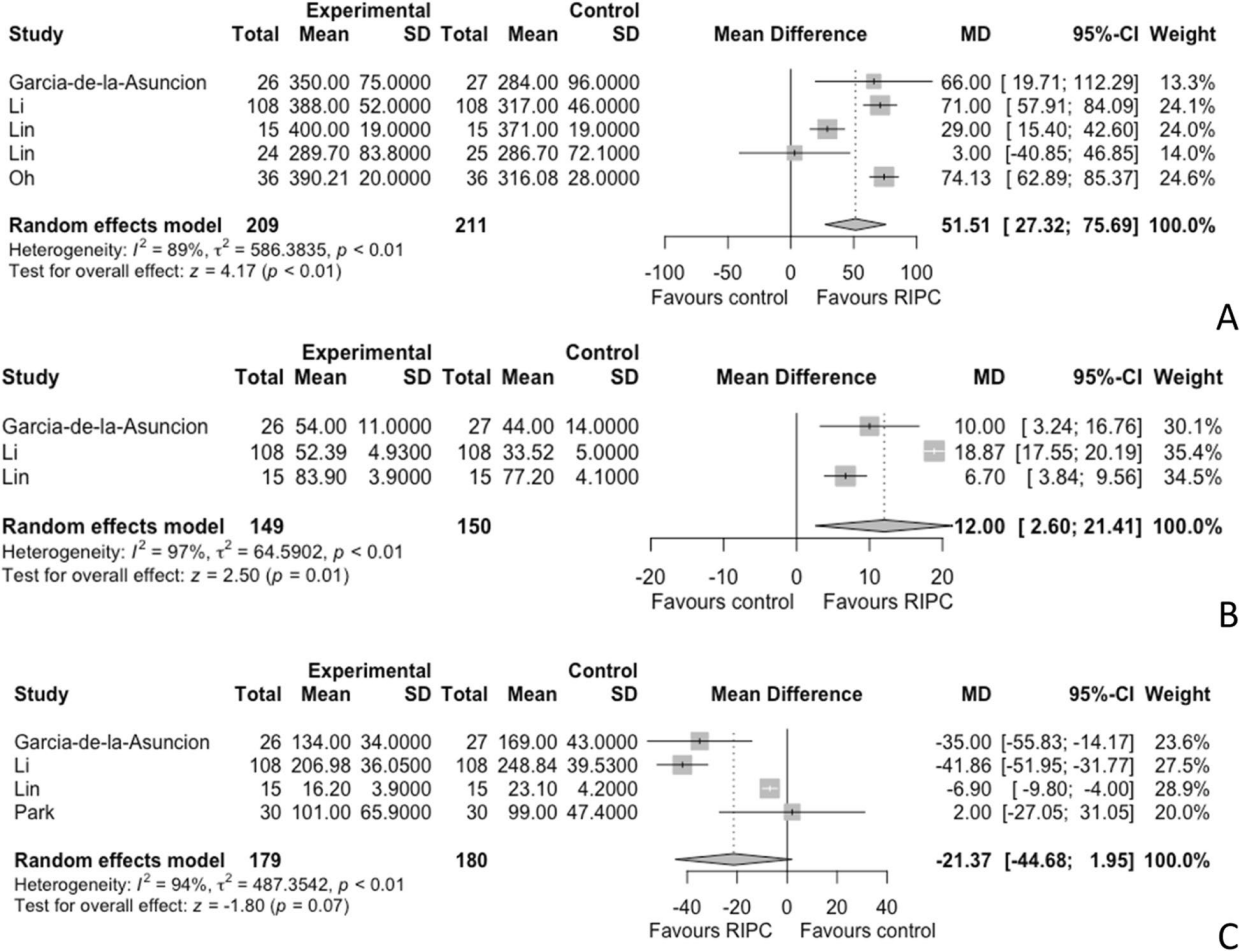
Meta-analysis comparing the effects of remote ischaemic preconditioning (RIPC) versus control on the ratio of arterial oxygen partial pressure to fractional inspired oxygen (P_a_O_2_/F_i_O_2_) within 6 h postoperatively (**A**), the arterial to alveolar partial pressure of oxygen (P_a_O_2_/P_A_O_2_) (**B**), and the alveolar-arterial partial pressure of oxygen difference (P_A_O_2_-P_a_O_2_) (**C**) in patients undergoing non-cardiac non-vascular surgery

Respiratory outcomes following the use of RIPC in NCNV surgery were published in 6 studies, and they included the ratio of arterial oxygen partial pressure to fractional inspired oxygen (P_a_O_2_/F_i_O_2_), the arterial to the alveolar partial pressure of oxygen (P_a_O_2_/P_A_O_2_), and the alveolar-arterial partial pressure of oxygen difference (P_A_O_2_-P_a_O_2_) (Park et al. 2018; Oh et al. 2017; Garcia-de-la-Asuncion et al. 2017; Li et al. 2014; Lin et al. 2010; Lin et al. 2014). Two of the studies examined lung lobectomy, 1 lung transplantation, and 3 orthopedic surgery. The P_a_O_2_/F_i_O_2_ within 6 h postoperatively was published in 5 studies and was significantly higher in the RIPC group, MD 51.51 mmHg, 95% CI 27.32 to 75.69, *p* < 0.01, *I*^2^ = 89% (Fig. 4A) (Oh et al. 2017; Garcia-de-la-Asuncion et al. 2017; Li et al. 2014; Lin et al. 2010; Lin et al. 2014). At 24 h postoperatively, the P_a_O_2_/F_i_O_2_ was also higher in the RIPC group, MD 26.56 mmHg, and 95% CI - 12.27 to 65.39, but the difference did not reach statistical significance (Garcia-de-la-Asuncion et al. 2017; Lin et al. 2010; Lin et al. 2014). Postoperative P_a_O_2_/P_A_O_2_ was published in 3 studies and was higher in the intervention group, MD 12, 95% CI 2.6 to 21.41, *p* < 0.01 (Fig. 4B) (Garcia-de-la-Asuncion et al. 2017; Li et al. 2014; Lin et al. 2010). Postoperative P_A_O_2_-P_a_O_2_ was published in 4 studies, three at 6 h postoperatively and one at the end of surgery (Park et al. 2018; Garcia-de-la-Asuncion et al. 2017; Li et al. 2014; Lin et al. 2010). There was no statistically significant difference between the intervention and control groups, MD - 21.37 mmHg, 95% CI - 44.68 to 1.95, and *p* = 0.07 (Fig. 4C).

### Postoperative liver function

**Fig. 5:**
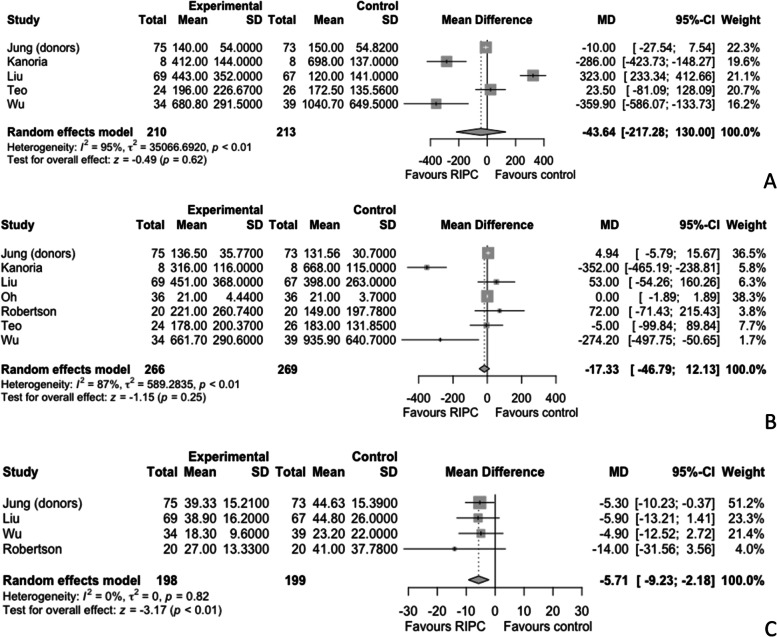
Meta-analysis comparing the effects of remote ischaemic preconditioning (RIPC) versus control on postoperative alanine aminotransferase (ALT) (**A**), aspartate aminotransferase (AST) (**B**), and bilirubin (**C**) in patients undergoing non-cardiac non-vascular surgery

Liver outcomes were published in 7 studies, of which 2 involved liver transplant surgery, 4 liver resections, and 1 orthopedic surgery (Robertson et al. 2017; Teo et al. 2020; Oh et al. 2017; Jung et al. 2020; Kanoria et al. 2017; Liu et al. 2019; Wu et al. 2020). Of the transplant studies, 1 applied RIPC to living donors and 1 to recipients (Robertson et al. 2017; Jung et al. 2020; Cordero-Pérez et al. 2018). ALT was available from 5 studies including patients undergoing liver resections (Teo et al. 2020; Jung et al. 2020; Kanoria et al. 2017; Liu et al. 2019; Wu et al. 2020). There was no difference in postoperative ALT between the control and intervention groups (MD - 43.64 IU/L, 95% CI - 217.28 to 130) (Fig. 5A). Postoperative AST was recorded from all 7 studies, and there was no difference between the control and intervention groups (MD - 17.33 IU/L, 95% -46.79 to 12.13) (Fig. 5B) (Robertson et al. 2017; Teo et al. 2020; Oh et al. 2017; Jung et al. 2020; Kanoria et al. 2017; Liu et al. 2019; Wu et al. 2020). Postoperative bilirubin was available from 4 studies, 3 related to liver resections and 1 to liver transplant recipients. Postoperative bilirubin was significantly lower in the RIPC group (MD -5.71 µmol/L, 95% CI -9.23 to -2.18, *p* < 0.01, I^2^ = 0) (Fig. 5C) (Robertson et al. 2017; Jung et al. 2020; Liu et al. 2019; Wu et al. 2020). A further randomised controlled study by Rakic et al. investigated the use RIPC in 60 patients undergoing liver resection and showed significantly lower postoperative bilirubin 17.5 vs 26.6 µmol/L, p = 0.031, AST 844.5 vs 978.3 IU/L, p = 0.021 and ALT 910.3 vs 1114.3 IU/L, *p* = 0.005 in the intervention vs the control group. However, we were unable to include the results in the meta-analysis because no measure of variation was reported (Rakic et al. 2018).

### Postoperative inflammatory and oxidative stress markers

Levels of Interleukin-6 (IL-6) were published in 9 studies (Zeggeren et al. 2021; Robertson et al. 2017; Oh et al. 2017; MacAllister et al. 2015; Li et al. 2014; Lin et al. 2010; Murphy et al. 2010; Oh et al. 2020; Elano et al. 2016). There was no statistically significant difference between the control and intervention groups (SMD - 0.46, 95% CI - 1.01 to 0.09) (Supplemental Fig. 2A). Postoperative tumor necrosis factor-a (TNF-a) was recorded in 5 studies. TNF-a was significantly lower in the intervention group, SMD - 0.82, 95% CI - 1.47 to - 0.18, *p* = 0.01, *I*^2^ - = 94% (Supplemental Fig. 2B) (Oh et al. 2017; MacAllister et al. 2015; Li et al. 2014; Cho et al. 2017; Tosun et al. 2021). Values of postoperative malondialdehyde (MDA) were available from 3 studies, and they were significantly lower in the RIPC group (MD - 1.24 µmol/L, 95% CI - 2.4 to - 0.07, *p* = 0.04, *I*^2^ = 91%) (Supplemental Fig. 2C) (Chen et al. 2013; Lin et al. 2010; Koca et al. 2011).

### Postoperative length of hospital stay

Fifteen studies evaluated the postoperative length of stay (Antonowicz et al. 2018; Ekeloef et al. 2019; Zeggeren et al. 2021; Robertson et al. 2017; Chung et al. 2021; Chen et al. 2013; Nicholson et al. 2015; MacAllister et al. 2015; Li et al. 2014; Lin et al. 2010; Jung et al. 2020; Liu et al. 2019; He et al. 2017; Krogstrup et al. 2017; Memtsoudis et al. 2014). There was a small but statistically significant difference favoring the intervention group, MD - 0.99 days, 95% CI - 1.75 to - 0.23, *p* = 0.01, *I*^2^ = 88% (Supplemental Fig. 3). The result potentially carries clinical significance, considering the bed availability constraints and healthcare costs.

### RIPC adverse events

Sixteen studies commented on the presence of adverse events related to RIPC (Antonowicz et al. 2018; Ekeloef et al. 2019; Zeggeren et al. 2021; Teo et al. 2020; Chung et al. 2021; Chen et al. 2013; Huang et al. 2013; Nicholson et al. 2015; MacAllister et al. 2015; Lin et al. 2014; Kanoria et al. 2017; Oh et al. 2020; Tosun et al. 2021; Krogstrup et al. 2017; Krag et al. 2019; Wang et al. 2018). Four studies identified adverse events, most of which were transient local erythema, petechiae, or bruising (Chen et al. 2013; Nicholson et al. 2015; MacAllister et al. 2015; Krogstrup et al. 2017). Chen et al. reported one patient with constriction-type feeling in the treated leg that was relieved after 1 day of physical therapy and Krogstrup et al. reported one adverse event in a patient in the control group due to machine malfunctioning resulting in uninterrupted inflation (Chen et al. 2013; Krogstrup et al. 2017).

A risk of bias analysis and justification for the studies included in the meta-analysis and a GRADE summary of findings are given in the supplemental material (Supplemental Fig. 4, Supplemental Table 2 and Supplemental Table 3).

## Discussion

We performed a systematic review of the literature and found inadequate evidence on the effect of RIPC on a postoperative myocardial injury. The definition of myocardial injury varied between the studies. Three studies reported the incidence of myocardial injury based on prespecified troponin values and showed promising results with a relative reduction in the incidence of postoperative myocardial injury ranging from 16 to 35%. Our review failed to show any significant difference in postoperative troponin levels, although only 4 studies were identified and there was significant statistical heterogeneity. The beneficial effect of RIPC on postoperative cardiac morbidity is supported by two recent systematic reviews in cardiac surgery by Benstoem et al. and Xie et al. that concluded that RIPC reduced the troponin release within the first 72 h after surgery (Benstoem et al. 2017; Xie et al. 2018). This finding is significant as several studies have shown that elevated troponin postoperatively is associated with an increased risk of mortality both in cardiac and non-cardiac surgery (Domanski et al. 2015; Devereaux et al. 2017). As the VISION study investigators showed, even mildly raised troponin levels were associated with at least 3 times increased risk of a 30-day mortality and the risk increased as the level of troponin increased (Devereaux et al. 2017).

Meta-analysis of the identified studies on renal outcomes showed moderate quality evidence of lower postoperative creatinine in the RIPC group, but no significant difference in either postoperative AKI or GFR. Although the clinical significance of a minimal reduction in postoperative creatinine is uncertain, it is well known that the change in serum creatinine is a rather poor and late indicator of renal dysfunction as it takes 24–72 h to reach levels diagnostic of AKI. Urinary biomarkers on the contrary are able to identify patients at risk of AKI within hours of the insult to the kidney. A meta-analysis of the effect of RIPC on postoperative NGAL showed low-quality evidence of lower NGAL levels in the RIPC group. NGAL is an early and sensitive marker for the development of AKI rising within hours of the renal insult; however, the cutoff value varies depending on the clinical setting and may be affected by several factors, including age, sepsis, and chronic kidney disease (Rizvi and Kashani 2017; Bennett et al. 2008). A meta-analysis of the use of RIPC in cardiac surgery by Deferrari et al. showed that RIPC significantly reduced the incidence of AKI in patients undergoing surgery maintained under volatile anesthesia (OR 0.57, 95% CI 0.41–0.79) but not in patients under propofol anesthesia (Deferrari et al. 2018). Zarbock et al. showed that the protective effect of RIPC against adverse renal events extended to 90 days postoperatively and the authors suggested that the failure of previous studies to show significant benefit is likely due to the use of propofol as well as the preferential beneficial effect of RIPC on high-risk patients (Zarbock et al. 2017). The clinical significance of our findings is unclear given the moderate and low-quality evidence from this meta-analysis. Additionally, both NGAL and postoperative creatinine may not be accurate indicators of renal function particularly in the perioperative setting. However, the potential of RIPC to protect from the development of AKI is important as AKI is often followed by the development of further complications and even mild (stage 1) postoperative AKI is associated with adverse long-term outcomes even when the renal function appears to have recovered prior to hospital discharge (Singbartl and Joannidis 2015; Mehta et al. 2011; Hobson et al. 2009).

Examining respiratory variables showed moderate quality evidence that RIPC has a beneficial effect on both P_a_O_2_/F_i_O_2_ and P_a_O_2_/P_A_O_2_. It has been proposed that impaired lung perfusion attributed to one-lung ventilation during lung resection surgery with subsequent increase in oxidative stress as well as a rise in inflammatory markers as a result of the surgical stress response contribute to postoperative acute lung injury (Garcia-de-la-Asuncion et al. 2017). Although P_a_O_2_/F_i_O_2_ is a surrogate endpoint of respiratory function, it has been shown, under standardized ventilator settings, to be predictive of mortality in patients with ARDS (Villar et al. 2015). In the perioperative setting Esteve et al. showed that P_a_O_2_/F_i_O_2_ < 242 at 3 h after cardiac surgery was associated with increased incidence of respiratory complications and P_a_O_2_/F_i_O_2_ < 202 was also associated with increased hospital mortality (Esteve et al. 2014). The effect of RIPC is potentially clinically significant particularly in patients with impaired baseline respiratory function.

Our meta-analysis on the effect of RIPC on hepatic outcomes showed moderate quality evidence of lower postoperative bilirubin but no difference in transaminases. A systematic review of the effect of local ischaemic preconditioning (Pringle maneuver) of the donor’s liver prior to transplantation by Robertson et al. in 2016 showed a reduction in liver injury, as indicated by lower AST level on day 3 and reduced 1-year mortality at 6 vs 11% (Robertson et al. 2016). However, a similar review on hepatectomies by Guo et al. in 2017 failed to show a significant difference in clinical or biochemical outcomes, although the authors suggested there may be some benefit in cirrhotic patients (Guo et al. 2017). An isolated lower postoperative bilirubin is an inadequate indicator of hepatic function and likely of small clinical significance.

Meta-analysis of the studies investigating oxidative stress and inflammatory markers showed lower TNF-a and MDA values postoperatively in the preconditioning group; however, the evidence was of low quality. Both TNF-a and MDA have been associated with increased delirium and cognitive decline postoperatively (Kazmierski and Kloszewska 2010; Wu et al. 2019; Terrando et al. 2010). Indeed, He et al. showed that RIPC improves the cognitive function of elderly patients undergoing bowel surgery (He et al. 2017). Despite this positive finding, oxidative stress markers are not currently used in clinical practice and remain largely experimental markers.

Finally, RIPC was associated with approximately 1 day less stay in the hospital, although the quality of the evidence was moderate as statistical heterogeneity (*I*^2^) was significant.

This is the first systematic review to assess the effect of RIPC in NCNV surgery. Some of the major limitations of our study are the significant degree of statistical heterogeneity and the small number of studies addressing certain outcomes. That likely stems from the diversity of surgical procedures included, which is expected given that the study of RIPC outside NCNV surgery is limited. Additionally, our meta-analysis includes data derived from graphs or calculated from published results (e.g., standard deviation from confidence interval) and that may have affected the accuracy of the results. Finally, our outcomes of interest include several surrogate endpoints that may not necessarily correlate well with clinical outcomes, e.g., P_a_O_2_/F_i_O_2_ vs need for respiratory support (Fleming and Powers 2012).

## Conclusion

In this systematic review and meta-analysis of the use of RIPC in non-cardiac non-vascular surgery, we found no evidence that RIPC affects postoperative troponin or AKI. There was inadequate evidence to conclude about the effect of RIPC on the perioperative myocardial injury.

We found moderate-quality evidence that RIPC is associated with lower postoperative creatinine and low-quality evidence of lower NGAL. We found moderate evidence that RIPC is associated with improvement in gas exchange based on P_a_O_2_/F_i_O_2_ and P_a_O_2_/P_A_O_2_. Similarly, moderate evidence was identified that RIPC is associated with a reduction in postoperative bilirubin and reduced length of hospital stay. We found low-quality evidence that RIPC is associated with lower TNF-a and MDA.

The results should be interpreted with caution as the heterogeneity was considerable, and most outcomes were addressed by a small number of studies. Further evidence on the use of RIPC in general surgery, particularly as regards to its effects on postoperative cardiac and renal morbidity including the relevant biomarkers would help clarify the role of this simple intervention in the prevention of postoperative morbidity.

## Supplementary Information

Additional file 1: **Supplemental Table 1.** Characteristics of the included studies alphabetically by author surname. **Supplemental Fig. 1.** Meta-analysis comparing the effects of Remote Ischaemic Preconditioning (RIPC) versus control on postoperative Glomerular Filtration Rate (GFR) in patients undergoing non-cardiac non-vascular surgery.** Supplemental Fig. 2.** Meta-analysis comparing the effects of Remote Ischaemic Preconditioning (RIPC) versus control on postoperative Interleukin 6 (IL-6) (A), Tumor Necrosis Factor a (TNF-a) (B) and Malondialdehyde (MDA) (C) in patients undergoing non-cardiac non-vascular surgery. **Supplemental Fig. 3.** Meta-analysis comparing the effects of Remote Ischaemic Preconditioning (RIPC**)** versus control on postoperative length of hospital stay.** Supplemental Fig. 4.** Risk of bias using the Revised Cochrane risk-of-bias tool for randomized trials (RoB 2). **Supplemental Table 2.** Risk of bias assessment details. **Supplemental Table 3.** GRADE Summary of findings table of the effects of Remote Ischaemic Preconditioning (RIPC**)** in non-cardiac non-vascular surgery.

## Data Availability

The datasets used and/or analysed during the current study are available from the corresponding author on reasonable request.

## Abbreviations

AKI: Acute kidney injury
ALT: Alanine aminotransferase
AST: Aspartate aminotransferase
CI: Confidence intervals
Cr-EDTA: Chrome-ethylenediamine tetraacetic acid
cTropI: Cardiac troponin I
GFR: Glomerular filtration rate
hs-TropI: High sensitivity troponin I
hs-TropT: High sensitivity troponin T
IL-6: Interleukin 6
MD: Mean difference
NCNV: Non-cardiac non-vascular
NGAL: Neutrophil gelatinase-associated lipocalin
PRISMA: Preferred Reporting Items for Systematic Reviews and Meta-Analyses
RIPC: Remote ischaemic preconditioning
RoB 2: Risk of bias tool 2
SMD: Standardised mean difference
99Tcm-DTPA: Technetium-diethylene triamine pentacetic acid
TNF-a: Tumour necrosis factor a
