# Supplementary material for: Efficacy of remote ischaemic preconditioning on outcomes following non-cardiac non-vascular surgery: a systematic review and meta-analysis
Source: Perioper Med (Lond). 2023 Apr 10;12:9. doi: 10.1186/s13741-023-00297-0 (PMC10084674; doi:10.1186/s13741-023-00297-0)
Supplement: Supplementary file 1 — Additional file 1: Supplemental Table 1. Characteristics of the included studies alphabetically by author surname. Supplemental Fig. 1. Meta-analysis comparing the effects of Remote Ischaemic Preconditioning (RIPC) versus control on postoperative Glomerular Filtration Rate (GFR) in patients undergoing non-cardiac non-vascular surgery. Supplemental Fig. 2. Meta-analysis comparing the effects of Remote Ischaemic Preconditioning (RIPC) versus control on postoperative Interleukin 6 (IL-6) (A), Tumor Necrosis Factor a (TNF-a) (B) and Malondialdehyde (MDA) (C) in patients undergoing non-cardiac non-vascular surgery. Supplemental Fig. 3. Meta-analysis comparing the effects of Remote Ischaemic Preconditioning (RIPC) versus control on postoperative length of hospital stay. Supplemental Fig. 4. Risk of bias using the Revised Cochrane risk-of-bias tool for randomized trials (RoB 2). Supplemental Table 2. Risk of bias assessment details. Supplemental Table 3. GRADE Summary of findings table of the effects of Remote Ischaemic Preconditioning (RIPC) in non-cardiac non-vascular surgery. [file 13741_2023_297_MOESM1_ESM.docx]

**Supplemental Table 1**. Characteristics of the included studies alphabetically by author surname

| Author | Year | Country | Surgery | Anaesthesia | RIPC protocol | Sample size (RIPC/Control) |
| --- | --- | --- | --- | --- | --- | --- |
| Antonowicz^1^ | 2018 | UK | Elective abdominal | Volatile or TIVA maintenance | Arm  200 mmHg  5min x 3 | 41/43 |
| Bang^2^ | 2019 | Korea | Renal transplant (RIPC in living donors, outcomes in donors and recipients) | Thiopentone induction and volatile maintenance | Arm  200 mmHg  5min x 3 | 85/85 donors  85/83 recipients |
| Chen^3^ | 2013 | China | Renal transplant (RIPC in donor or recipient, outcomes in donors and recipients) | Not specified | Thigh  300 mm Hg  5min x 3 | 20/20 |
| Chung^4^ | 2021 | Korea | Nephrectomy | Volatile maintenance | Arm  250 mmHg  5min x 4 | 41/40 |
| Ekeloef^5^ | 2019 | Denmark | Hip fracture | Regional, volatile or TIVA | Arm  200 mmHg  5min x 4 | 286/287 |
| Garcia-de-la-Asuncion^6^ | 2017 | Spain | Pulmonary lobectomy | Epidural, thiopentone induction and volatile maintenance | Thigh  200 mmHg  5min x 3 | 26/27 |
| He^7^ | 2017 | China | Open colectomy | Midazolam, etomidate induction and volatile maintenance | Arm  200 mmHg  5min x 3 | 45/45 |
| Hou^8^ | 2017 | China | Laparoscopic partial nephrectomy | Midazolam, etomidate induction and TIVA maintenance | Arm  200 mmHg  5min x 3 | 20/20 |
| Hu^9^ | 2010 | China | Cervical spine decompression | TIVA | Arm  200 mmHg  5min x 3 | 20/20 |
| Huang^10^ | 2013 | China | Laparoscopic partial nephrectomy | Not specified | Thigh  200 mmHg  5min x 3 | 40/38 |
| Jung^11^ | 2018 | Korea | Liver transplant (RIPC in living donor, outcomes in donors and recipients) | Propofol induction, volatile maintenance | Arm  200 mmHg  5min x 3 | 75/73 |
| Kanoria^12^ | 2017 | UK | Liver resection | Not specified | Thigh  Systolic x 2  10min x 3 | 8/8 |
| Koca^13^ | 2019 | Turkey | Arthroscopic knee surgery | Spinal | Thigh  350 mmHg  5min x 3 | 15/15 |
| Krag^14^ | 2017 | Denmark | Head and neck cancer surgery | Not specified | Arm  200 mmHg  5min x 4 | 30/30 |
| Krogstrup^15^ /Nielsen^16^ | 2017 | Denmark | Renal transplant (RIPC in recipients) | Propofol induction, volatile maintenance | Thigh  250 mmHg  5min x 4 | 109/113 |
| Li^17^ | 2014 | China | Pulmonary resection | TIVA and epidural | Arm  200 mmHg  5min x 3 | 108/108 |
| Lin^18^ | 2010 | China | Lower limb surgery | CSE | Thigh  Not specified  5min x 3 | 15/15 |
| Lin^19^ | 2014 | Australia | Lung transplant | TIVA | Thigh  300 mmHg | 30/30 |
| Liu^20^ | 2019 | China | Liver resection | Etomidate, midazolam induction, volatile maintenance | Arm  225 mmHg  5min x 3 | 69/67 |
| MacAllister^21^ | 2015 | Multicentre | Renal transplant (RIPC in donor and recipient, outcomes in recipients) | Not specified | Arm  Systolic+40 mmHg  5min x 4 | 98/93 |
| Memtsoudis^22^ | 2014 | US | Knee arthroplasty | CSE, regional and midazolam, propofol sedation | Thigh  250 mmHg  5min x 1 | 30/30 |
| Murphy^23^ | 2010 | Ireland | Knee arthroplasty | Spinal, not specified if any sedation | Thigh  Systolic+100 mmHg  5min x 3 | 10/10 |
| Nicholson^24^ | 2015 | UK | Renal transplant (RIPC in recipient, outcomes in recipients) | Volatile maintenance | Thigh  200 mmHg  5min x 4 | 40/40 |
| Oh^25^ | 2017 | Korea | Knee arthroplasty | Volatile maintenance | Thigh  Systolic x 2  5min x 3 | 36/36 |
| Oh C S^26^ | 2019 | Korea | Shoulder surgery | Volatile maintenance | Thigh  Systolic x 2  5min x 3 | 29/34 |
| Park^27^ | 2018 | Korea | Elective orthopaedic surgery | Spinal or volatile maintenance | Arm or calf  250 mmHg  5min x 3 | 30/30 |
| Pereira^28^ | 2016 | Brazil | Cholecystectomy | Spinal | Thigh  Systolic+100 mmHg  5min x 1 | 10/10 |
| Porpiglia^29^ | 2018 | Italy | Partial nephrectomy | Not specified | Arm  Not specified  5min x 4 | 25/21 |
| Rakic^30^ | 2018 | Croatia | Liver resection | Not specified | Arm  200 mmHg  5min x 3 | 20/20 |
| Robertson^31^ | 2017 | UK | Liver transplant (RIPC in recipients, outcomes in recipients) | Not specified | Thigh  200 mmHg  5min x 3 | 20/20 |
| Sullivan^32^ | 2009 | Ireland | Cruciate ligament reconstruction | Propofol induction, volatile maintenance | Thigh  250 mmHg  5min x 3 | 12/13 |
| Teo^33^ | 2020 | Singapore | Liver resection | Propofol induction, volatile maintenance | Arm  200 mmHg  5min x 4 | 24/26 |
| Tosun^34^ | 2021 | Turkey | Liver transplant (RIPC in recipients) | Not specified | Arm  250 mmHg  3min x 3 | 10/11 |
| Van^35^ | 2008 | Turkey | Lower limb surgery | CSE | Thigh  300 mmHg  5min x 3 | 10/10 |
| Van Zeggeren^36^ | 2021 | Netherlands | Pancreatic surgery | Volatile maintenance | Arm  200 mmHg  5min x 3 | 45/45 |
| Wu^37^ | 2020 | China | Liver resection | TIVA | Arm  200 mmHg  5min x 3 | 34/39 |


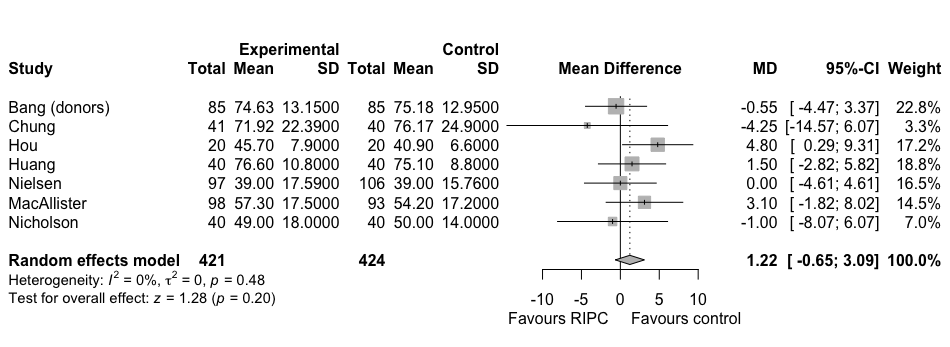
 A

Supplemental Figure 1. Meta-analysis comparing the effects of Remote Ischemic Preconditioning (RIPC) versus control on postoperative Glomerular Filtration Rate (GFR) in patients undergoing non-cardiac non-vascular surgery


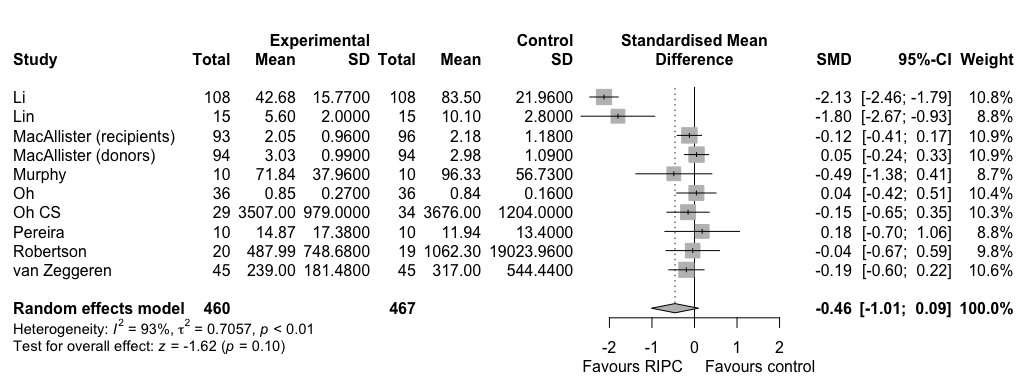


A


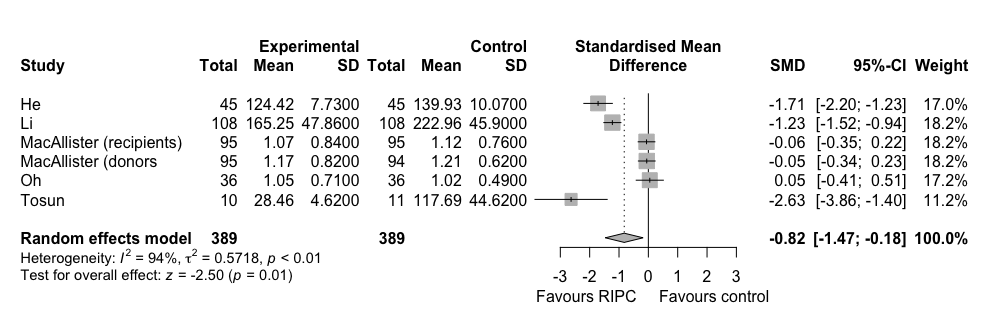
B


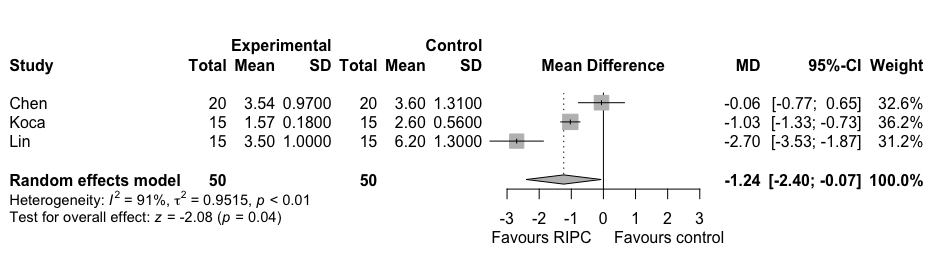


C

Supplemental Figure 2. Meta-analysis comparing the effects of Remote Ischemic Preconditioning (RIPC) versus control on postoperative Interleukin 6 (IL-6) (A), Tumor Necrosis Factor α (TNF-α) (B) and Malondialdehyde (MDA) (C) in patients undergoing non-cardiac non-vascular surgery.


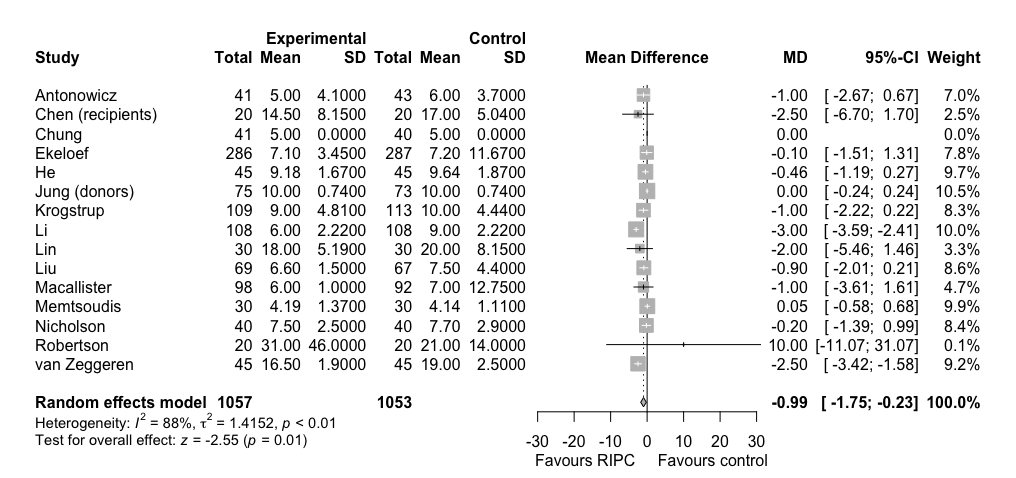


Supplemental Figure 3. Meta-analysis comparing the effects of Remote Ischemic Preconditioning (RIPC) versus control on postoperative length of hospital stay


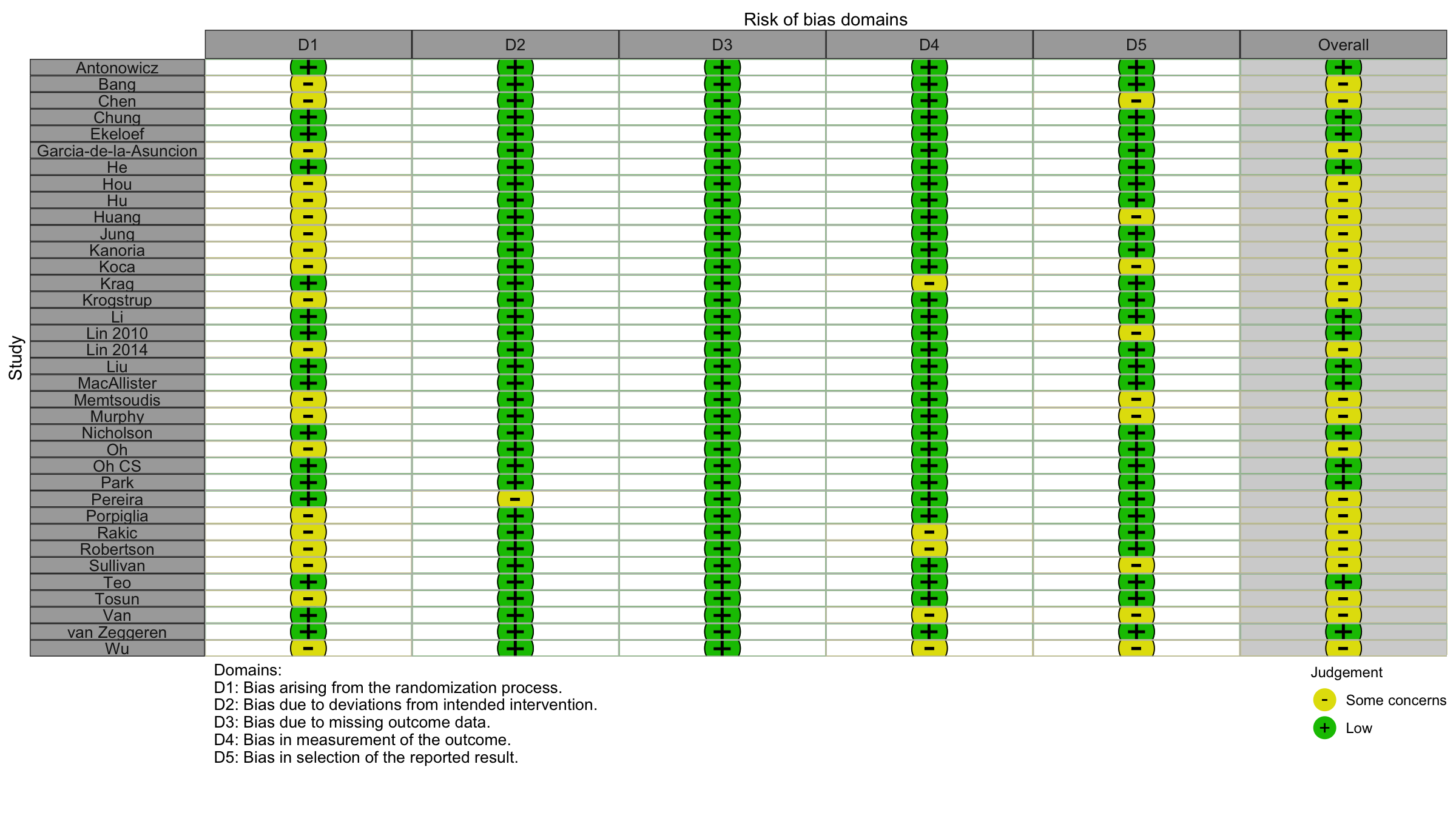


Supplemental Figure 4. Risk of bias using the Revised Cochrane risk-of-bias tool for randomized trials (RoB 2)

Supplemental Table 2. Risk of bias assessment details

| Author | Year | Country |
| --- | --- | --- |
| Antonowicz^1^ | 2018 | Nil |
| Bang^2^ | 2019 | Unclear allocation concealment |
| Chen^3^ | 2013 | Unclear allocation concealment, protocol not mentioned or found |
| Chung | 2021 | Nil |
| Ekeloef^5^ | 2019 | Nil |
| Garcia-de-la-Asuncion^6^ | 2017 | Allocation concealment and baseline balance NI |
| He^7^ | 2017 | Nil |
| Hou^8^ | 2017 | Unclear allocation concealment |
| Hu^9^ | 2010 | Unclear allocation concealment |
| Huang^10^ | 2013 | Allocation concealment, blinding of outcome assessors and protocol NI |
| Jung^11^ | 2018 | Baseline transaminases lower in RIPC group in donors and recipients |
| Kanoria^12^ | 2017 | Unclear allocation concealment |
| Koca^13^ | 2019 | Unclear allocation concealment, personnel unblinded but deviations unlikely, protocol not mentioned |
| Krag^14^ | 2017 | Outcome assessors. Not blinded and deviations NI |
| Krogstrup^15^ /Nielsen^16^ | 2017 | Unclear allocation concealment, unclear blinding of outcome assessors but deviations unlikely |
| Li^17^ | 2014 | Nil |
| Lin^18^ | 2010 | Protocol not mentioned |
| Lin^19^ | 2014 | Allocation concealment unclear |
| Liu^20^ | 2019 | Nil |
| MacAllister^21^ | 2015 | Nil |
| Memtsoudis^22^ | 2014 | Unclear allocation concealment, protocol NI |
| Murphy^23^ | 2010 | Unclear allocation concealment, unclear blinding of outcome assessors but deviations unlikely, protocol NI |
| Nicholson^24^ | 2015 | Unclear blinding of outcome assessors but deviations unlikely |
| Oh^25^ | 2017 | Unclear allocation concealment |
| Oh CS | 2019 | Nil |
| Park^27^ | 2018 | Nil |
| Pereira^28^ | 2016 | The patients were not blinded |
| Porpiglia^29^ | 2018 | Unclear allocation concealment, blinding unclear but deviations unlikely |
| Rakic^30^ | 2018 | Unclear allocation concealment, blinding of outcome assessors unclear and assessment could have been influenced though probably not. |
| Robertson^31^ | 2017 | Unclear allocation concealment, blinding of outcome assessors unclear and could have been influenced but probably not. |
| Sullivan^32^ | 2009 | Unclear allocation concealment and protocol not mentioned |
| Teo^33^ | 2020 | Nil |
| Tosun | 2021 | Randomization method unclear |
| Van^35^ | 2008 | Outcome assessors not blinded and assessment could have been influenced but probably not, protocol not mentioned |
| Van Zeggeren |  | Nil |
| Wu^37^ | 2020 | Unclear allocation concealment, blinding not mentioned and assessment could have been influenced though probably not, protocol, not mentioned |

Supplemental Table 3. GRADE Summary of findings table of the effects of Remote Ischemic Preconditioning (RIPC) in non-cardiac non-vascular surgery

| **Certainty assessment** | | | | | | | | | | | | | | | **№ of patients** | | | | | | | **Effect** | **Certainty** |
| --- | --- | --- | --- | --- | --- | --- | --- | --- | --- | --- | --- | --- | --- | --- | --- | --- | --- | --- | --- | --- | --- | --- | --- |
| **№ of studies** | **Study design** | **Risk of bias** | | **Inconsistency** | | | | **Indirectness** | | | | | **Imprecision** | | **[RIPC]** | | | | | | **[no RIPC]** | **Absolute (95% CI)** |  |
| **Postoperative Creatinine** | | | |  | | | | |  | | | | |  |  |  |  |  |  |  |  |  |  |
| 9 | randomised trials | serious ^a^ | | not serious | | | | not serious | | | | | not serious | | 454 | | | | | | 460 | MD **3.81 μmol/L lower** (6.79 lower to 0.83 lower) | ⨁⨁⨁◯ MODERATE |
| **NGAL** | |  |  | | | |  | | | | |  | | | | | |  | |  |  |  |  |
| 5 | randomised trials | not serious | | serious ^b^ | | | | not serious | | | | | serious ^c^ | | 188 | | | | | | 191 | SMD **0.66 SD lower** (1.27 lower to 0.06 lower) | ⨁⨁◯◯ LOW |
| **P/F ratio** | |  |  | | |  | | | | |  | | | | | |  | |  |  |  |  |  |
| 5 | randomised trials | not serious | | serious ^b^ | | | | not serious | | | | | not serious | | 209 | | | | | | 211 | MD **51.51 mm Hg higher** (27.32 higher to 75.69 higher) | ⨁⨁⨁◯ MODERATE |
| **Bilirubin** | |  |  | | |  | | | | |  | | | | | |  | |  |  |  |  |  |
| 4 | randomised trials | not serious | | not serious | | | | not serious | | | | | serious ^e^ | | 198 | | | | | | 199 | MD **5.71 μmol/L lower** (9.23 lower to 2.18 lower) | ⨁⨁⨁◯ MODERATE |
| **Length of stay** | |  |  | | |  | | | | |  | | | | | |  | |  |  |  |  |  |
| 15 | randomised trials | not serious | | serious ^b^ | | | | not serious | | | | | not serious | | 1057 | | | | | | 1053 | MD **0.99 days lower** (1.75 lower to 0.23 lower) | ⨁⨁⨁◯ MODERATE |
| **Tumour Necrosis Factor alpha (TNF-α)** | | | | |  | | | | |  | | | | | |  | | |  |  |  |  |  |
| 5 | randomised trials | serious ^f^ | | serious ^d^ | | | | not serious | | | | | not serious | | 389 | | | | | | 389 | SMD **0.82 SD lower** (1.47 lower to 0.18 lower) | ⨁⨁◯◯ LOW |

**CI:** Confidence interval; **OR:** Odds ratio; **MD:** Mean difference; **SMD:** Standardised mean differencea. Bang unclear allocation concealment and baseline imbalance with Creatinine lower in intervention group, Chen, Huang, Nielsen and Oh unclear allocation concealment, Nicholson unclear blinding of outcome assessors b. I2 >80% c. using the control values from Porpiglia, and assuming a reduction of 20% is significant, then OIS is 636>sample size d. I2>90% e. Using the control data from Wu's study and assuming that a 20% reduction in the intervention group would be significant then OIS is 608>sample size f. Oh unclear allocation concealment, Tosun unclear randomisation technique
